# Unresolved Issues Associated with Transcranial Magnetic Stimulation (TMS) Treatment of Chronic Tinnitus

**DOI:** 10.3390/jcm12144648

**Published:** 2023-07-12

**Authors:** Robert L. Folmer

**Affiliations:** 1 Department of Otolaryngology, Oregon Health & Science University, Portland, OR 97239, USA; robert.folmer@va.gov; 2 National Center for Rehabilitative Auditory Research (NCRAR), VA Portland Medical Center, Portland, OR 97239, USA

**Keywords:** tinnitus, transcranial magnetic stimulation, TMS, rTMS, efficacy

## Abstract

Transcranial magnetic stimulation (TMS) has been investigated as a potential treatment for chronic tinnitus for 20 years. Numerous studies have reported that repetitive TMS (rTMS) has demonstrated efficacy for reducing the severity of tinnitus and its associated co-conditions such as depression, anxiety, and insomnia. However, some researchers have reported that active rTMS is no more effective than sham (placebo) rTMS as a tinnitus treatment method. There are numerous unresolved issues in this field that need to be addressed before rTMS can become a viable treatment for tinnitus. These issues include the type or brand of TMS system and its configuration; coil type, orientation, and placement method; scalp or neural target; laterality of rTMS application; dual site vs. single site stimulation; stimulation frequency and intensity; number of sessions; number of pulses per session; determination of the resting motor threshold (rMT); characteristics of the study population and their tinnitus; and outcome measures and follow-up assessments. To address and resolve these issues, large-scale, multi-site clinical trials of rTMS for tinnitus need to be conducted to determine which rTMS protocols are the most effective. In the absence of such investigations, the issues that need to be studied and addressed remain unresolved and continue to impede the clinical application of this treatment method.

## 1. Introduction

Chronic tinnitus is a condition that negatively impacts the quality of life of millions of people worldwide [1,2,3,4,5,6,7,8,9,10,11]. Unfortunately, the number of people suffering from tinnitus continues to increase annually. The severity of tinnitus—that is, the negative impact the condition has on patients’ quality of life—is positively correlated with the severity of depression [1], insomnia [2], anxiety [3], and obsessive compulsiveness [7] experienced by individuals. Behavioral interventions and sound therapies (including hearing aids and sound generators) provide relief for some tinnitus sufferers [5,12], but a safe and effective physiological/medical treatment for tinnitus has been sought for decades, with mixed success at best [10]. A true “cure” for the most common etiologies of tinnitus remains elusive.

**Figure 1:**
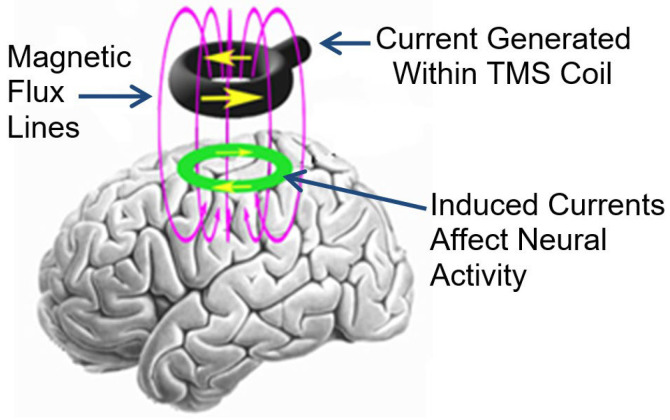
Illustration of how TMS delivered to the scalp affects the underlying neural tissue.

Since 2003, transcranial magnetic stimulation (TMS) has been investigated as a potential treatment for chronic tinnitus [13,14,15,16,17,18,19]. TMS is a non-invasive intervention that delivers electromagnetic pulses through a coil to the patient’s scalp. Ultimately, some of this energy is transmitted through the skull and affects the activity of the underlying neural tissue (see Figure 1 below).

**Figure 2:**
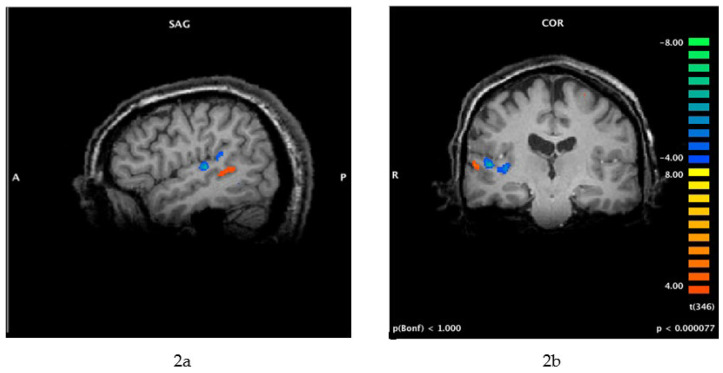
(**a**,**b**) Functional MRI (fMRI) of brain activity associated with tinnitus (this patient perceived 12,000 Hz tinnitus on the right side only). Cortical activity associated with tinnitus perception (orange) is more lateral (within secondary auditory cortex) compared with the neural activity in the primary auditory cortex that is associated with the perception of external sounds (blue). These images were obtained using a protocol that included residual inhibition, as follows: The scan began with a 30 s resting baseline, followed by 1 min of masking noise. The patient pressed one button when he perceived that his tinnitus was effectively masked, another button when his tinnitus began to return after masking, and a third button when his tinnitus was back to its original loudness. SAG = sagittal view; COR = coronal view; A = anterior; P = posterior; R = right.

Because several functional imaging studies have shown that people who experience tinnitus exhibit abnormal activity in the auditory cortex [20,21,22] and associated limbic regions of the brain [23,24,25,26,27], the neural mechanisms of tinnitus make the condition a good candidate for suppression by TMS (see Figure 2a,b below). 

Repetitive TMS (rTMS) has demonstrated effectiveness as a treatment for chronic tinnitus [13,14,15,16,17,18,19,28]. In their systematic review and meta-analysis of rTMS treatment studies for tinnitus, Soleimani et al. [16] concluded that all of the studies they included reported significant medium-to-large effect sizes, which provide evidence for the clinical efficacy of rTMS. However, Soleimani et al. stated that the high variability in study design and reported outcomes underscore the need to replicate findings with a large number of patients and long-term follow up. Folmer et al. [28], Langguth et al. [29], Piccirillo [30], Mennemeier and George [31], Liang et al. [17], Chen et al. [18], Yin et al. [19], and many other authors and researchers agree with this sentiment. Unfortunately, large, multi-site clinical trials of rTMS for tinnitus have, for the most part, not occurred. Part of the explanation for this void probably involves published studies that did not report efficacy of rTMS for tinnitus treatment [32,33]. Some of the reasons for these disparate findings include the unresolved issues in this field, which are the subject of this article.

## 2. Materials and Methods

The PubMed and Medline databases (National Center for Biotechnology Information, U.S. National Library of Medicine) were searched for the terms repetitive transcranial magnetic stimulation, tinnitus, TMS, and rTMS in articles published from 2003 to 2023. Publications mentioned in this article reflect some of the important variables to consider when conducting TMS studies for tinnitus, namely the use of a control/placebo condition, TMS stimulation parameters (intensity, frequency, number of pulses administered per session, and number of sessions), tonic versus burst stimulation, placement of the TMS coil (including laterality of stimulation and single site vs. multi-site stimulation), characteristics of the tinnitus population, and sample size.

## 3. Results

As stated in the Introduction, rTMS has demonstrated effectiveness as a treatment for chronic tinnitus in numerous studies and clinical trials [13,14,15,16,17,18,19,28], but some researchers have reported findings that are not as encouraging [32,33]. Some unresolved issues in this field are discussed below—these issues should be addressed in future studies so that rTMS might achieve its potential as a viable treatment for tinnitus.

Unresolved Issues Associated with rTMS Treatment of Chronic Tinnitus

### 3.1. rTMS Equipment

- Type/Brand of TMS System and Its Configuration.

Several different brands or types of TMS systems are commercially available for research and clinical applications. For example, Landgrebe et al. [33] used MagPro (Medtronic) stimulators and coils for their rTMS study involving tinnitus patients. Folmer et al. [28] used Magstim Rapid2 stimulators and Air Film coils in their tinnitus clinical trial. It is likely that the depth of penetration, size, and shape of the magnetic fields produced by these systems are different, and these factors might affect the treatment outcomes. Schoisswohl et al. [34] compared the MagPro and Magstim brands of TMS systems and found that the MagPro system induces current flow in an anterior−posterior to posterior−anterior direction (AP-PA), whereas the Magstim system produces an induced posterior−anterior to anterior−posterior current direction (PA-AP) in the brain. This might be part of the explanation for the positive clinical trial results reported by Folmer et al. [28] compared with the lower level of efficacy reported by Landgrebe et al. [33], even though the scalp target and other stimulation parameters were similar for the two studies. Schoisswohl et al. [34] concluded that the technical TMS parameter of the current direction might be essential for the efficacy of rTMS as a treatment for tinnitus. The authors stated “Systematic investigations of technical TMS parameters like current direction in larger samples of tinnitus patients are highly needed”.

- Coil Type

The majority of published studies of rTMS for tinnitus used figure-of-eight coils to deliver electromagnetic stimulation to the participant’s head. However, as described in the section above, different brands and configurations of coils might cause variations in the stimulation parameters that can affect treatment outcomes. While figure-of-eight coils stimulate a somewhat narrow region of cortex, newer H-coils can stimulate a wider and deeper neural area, and should be studied in tinnitus populations [35]. For a review of TMS coil types, see Ilmoniemi et al. [36].

- Coil Orientation

Many published studies of rTMS for tinnitus do not specify how their figure-of-eight coils were oriented during rTMS administration. Folmer et al. [28] positioned the TMS coil with the cord pointing downward. Differences in coil orientation might affect the induced current and its effect on neural activity and treatment outcomes. This detail should be specified in experimental protocols and studied in future investigations.

### 3.2. Coil Placement/Scalp Target

- Temporal Lobe Stimulation

The original studies of TMS for tinnitus targeted the temporal lobe because functional imaging indicated that the auditory cortex is one of the generators of tinnitus perception [15,16,17,20,21,22,37]. Therefore, Heschl’s gyrus was targeted with 1 Hz rTMS, with the goal of suppressing neural activity in the primary auditory cortex, which generates or contributes to the perception of tinnitus. However, as shown in Figure 2a,b, the primary auditory cortex (blue regions) is probably too deep to be affected by standard TMS systems and figure-of-eight coils. Figure 2a,b shows that the perception of tinnitus—for this individual, at least—is associated with activity in a neural region that is more superficial (orange region) and could be affected by standard rTMS methods. It is possible that improvements in tinnitus following a course of rTMS, as reported by Folmer et al. [28] and others, might result from the suppression of this neural region and/or disruption of the neural activity between this area and limbic regions that mediate patients’ negative reactions to tinnitus (tinnitus severity) [18,19,20,21,22,23,24,25,26,27]. Other studies of rTMS for tinnitus targeted the temporoparietal junction [32,38]—which is slightly posterior to Heschl’s gyrus—with limited success.

- Neuronavigation vs. Coil Placement via Scalp Landmarks

Langguth et al. [37], Folmer et al. [28], and others used the international 10–20 EEG electrode placement system and scalp landmarks to position the TMS coil on the patient’s head. Other researchers used functional and/or anatomical magnetic resonance imaging (MRI) in combination with neuronavigational systems to identify neural targets and position the TMS coil [39]. However, because the optimal neural target for rTMS in tinnitus patients has not been definitively identified, it is difficult to discern if these neuronavigational methods are worth the extra time and expense they require. In addition, as rTMS stimulates relatively diffuse neural regions, pinpointing the placement of the TMS coil might not be necessary or warranted. Noh et al. [39] compared the 10–20 coil placement method with neuronavigation-guided coil placement in a population of tinnitus patients and concluded, “We found that the treatment outcomes were similar for both the10–20 EEG system-based rTMS and neuronavigation-based rTMS. It seems that the method of target localization is not a critical factor in the treatment outcome of rTMS when treating tinnitus patients.”

- Frontal/Dual Site Stimulation

While early studies of rTMS for tinnitus targeted the temporal lobe, more recent studies delivered stimulation to two neural sites in succession: the frontal lobe and the temporal lobe. For example, Langguth et al. [40] delivered 20 Hz rTMS over the left frontal cortex, followed by 1 Hz rTMS over the left auditory cortex of tinnitus patients. They reported that the percentage of treatment responders was higher for the combined frontal and temporal rTMS (43%) compared with the sham rTMS condition (6%). However, Formanek et al. [41] observed “no significant effect of bilateral low-frequency rTMS of the primary auditory cortex and high-frequency stimulation of the left dorsolateral prefrontal cortex” in a group of 20 tinnitus patients. More recently, Noh et al. [42], Poeppl et al. [43], and Marder et al. [44] all reported that dual site rTMS demonstrated efficacy for tinnitus. Noh et al. [42] and Marder et al. [44] concluded that dual site stimulation was superior to single site rTMS for tinnitus.

- Laterality of rTMS Application

Some researchers (e.g., Landgrebe et al. [33]) administered rTMS to only the left side of the head for all participants. Participants in Folmer et al.’s clinical trial [28] were randomized to receive rTMS to either the left or right side of the head. Folmer [22] and other researchers demonstrated that the right auditory cortex sometimes exhibits superfluous activity associated with tinnitus perception (see Figure 2a,b). Frank et al. [45] reported that patients who perceived tinnitus primarily on the right side did not benefit from left-side rTMS. In light of these findings, protocols for tinnitus that administer rTMS to the left side of the head only should be re-evaluated. Right-side rTMS might benefit some tinnitus patients who do not respond to left-side stimulation. Marder et al. [44] delivered rTMS to 10 tinnitus patients in a sequential protocol: (1) excitatory stimulation administered to the left dorsolateral prefrontal cortex (DLPFC) or inhibitory stimulation administered to the right DLPFC, followed by (2) inhibitory stimulation administered to Heschel’s gyrus. The results indicated that the addition of 1Hz rTMS at the right DLPFC was superior to single site rTMS in the short term (1–12 weeks), while the addition of 20 Hz rTMS at the left DLPFC appeared superior in the long term (90–180 days). 

### 3.3. Stimulation Parameters

- Stimulation Frequency and Number of Pulses Per Session

The stimulation frequency of 1 Hz is often used to deliver rTMS to the temporal lobe because this frequency is thought to suppress neural activity in the region beneath the coil [46]; 1500–2000 rTMS pulses are sometimes administered at this stimulation rate per session [15]. However, other stimulation rates have also been investigated [15]. Ring et al. [47] administered alpha burst rTMS to 23 military veterans who experienced tinnitus. Five rTMS pulses (frequency = 25 Hz) were delivered with an inter-burst interval of 100 ms. This burst pattern was repeated for five iterations (a total of 1500 ms) followed by a 13 s rest period, then another train of bursts. Forty burst trains were administered for a total of 5 min of stimulation per application. The protocol was applied first over the right temporal region (EEG electrode position T4), then repeated over the left temporal lobe (EEG electrode position T3). Alpha burst stimulation (ABS) was applied for 10 daily sessions. Ring et al. [47] reported that 18 of the 23 study participants experienced significant improvements in tinnitus symptoms post-treatment. 

Continuous theta burst stimulation (cTBS) is another rTMS protocol that has been investigated with tinnitus patients. Hong et al. [48] conducted a pilot study of cTBS involving 15 tinnitus patients and administered stimulation according to the following parameters: One session of continuous theta-burst stimulation (cTBS) involved 3 TMS pulses of 50 Hz (i.e., 20 ms between each stimulus) repeated at a 200 ms interval (i.e., 5 Hz) for 20 s at a stimulus intensity of 70% rMT (resting motor threshold). They applied 4 sessions at a 1 s interval, and after 15 min, another 4 sessions with a 1 s gap between sessions, per day (2400 pulses/day) for 5 consecutive days. However, no significant therapeutic effect was observed in the small treatment group. Godbehere [49] conducted a cTBS study involving 40 tinnitus patients and administered stimulation according to this protocol: Each stimulation train (40 s) consisted of 600 stimuli applied in bursts of 3 pulses at 50 Hz given every 200 ms (5 Hz). A rest period of 15 min was provided between the first and second trains. The actual or sham treatments were provided on 5 consecutive days. Patients in both the active and sham stimulation groups exhibited improvements in tinnitus severity, but there was no significant difference in results between the two study arms.

- Number of Sessions

Some of the early rTMS studies of tinnitus administered 1 Hz stimulation for 5 consecutive days [50,51]; others did so for 10 consecutive week days [37,52]. Some of the more recent cTBS studies provided 5 days of stimulation per patient [48,49]. The original clinical application of rTMS—for major depression—often includes a course of daily treatment sessions that are conducted for 3–6 weeks or more [53,54]. This number of treatment sessions has rarely been investigated in tinnitus patients. Recently, Cole et al. [55] reported that an accelerated form of intermittent theta-burst stimulation (iTBS) was effective for reducing depression after a treatment course of 5 days. This required 10 treatment sessions per day; however, and a total of 18,000 pulses per day were administered to each patient.

- Stimulation Intensity and Determination of Resting Motor Threshold (rMT).

Resting motor threshold (rMT) is determined for TMS studies to calibrate the stimulation intensity for patients as a function of their rMT. Landgrebe et al. [33] administered rTMS at 110% of each participant’s rMT. However, they did not specify how rMT was determined. The mean rTMS intensity (57% of Magstim system capacity) used for the active stimulation group in Folmer’s study [28] corresponded to a magnetic field strength of approximately 0.6 T at the surface of the coil. If this rTMS intensity was greater than that used by Landgrebe et al. [33] or Hong et al. [48], it could have contributed to differences in results among the studies. Because the methods used to determine rMT varied from study-to-study, and from researcher-to-researcher, it was difficult to compare the stimulation intensities between studies. However, this is an important variable because stimulation intensity is likely to influence the extent of neural activation/suppression, and thus to affect treatment outcomes.

- Placebo/Sham Control Condition and Blinding

Studies of rTMS for tinnitus that do not include a placebo/sham control condition are problematic because the placebo effect among tinnitus patients is often significant [56]. Placebo (or ‘‘sham’’) procedures that have been used in rTMS tinnitus studies include recordings of active stimulation [57], tilting the coil 45 or 90 degrees [58], stimulating a non-target region of scalp [59], or using a placebo coil that seems identical to the active coil. Vanneste et al. [60] used a Magstim placebo coil and wrote, “As the sham coil only mimics the sound of active TMS but lacks the somatosensory sensation, it is not an optimal control condition” (p. 1146). Therefore, rTMS crossover designs for TMS studies are of limited value. 

Blinding is another significant challenge in rTMS studies because clinicians or technicians delivering treatment usually know when active or placebo stimulation is being administered. In most studies, it was also easy for research subjects to distinguish between active and placebo rTMS conditions. For this reason, Folmer et al. [28] used a parallel study design, instead of a crossover design. The Magstim sham Airfilm coil used in their study was identical in appearance to the active Magstim Airfilm coil. The sham coil also generated sounds that were similar to the active coil and produced tapping sensations on the subject’s scalp that were similar to those produced by the active coil. At the conclusion of the 10th (and last) rTMS session in Folmer et al.’s clinical trial [28], each subject was asked to guess if he/she had received active or placebo stimulation during the trial. The results shown in Table 1 demonstrate that subjects’ guesses were no better than chance, indicating that the placebo coil was an effective control. 

The fact that a majority of subjects (42 of 64) guessed “placebo rTMS” probably reflects the expectation of some patients that their tinnitus would be eradicated or greatly reduced in volume following 10 sessions of active rTMS. If this did not happen, research participants were more likely to guess that they were in the placebo group. 

Keller [61] suggested that participants in rTMS studies should be asked whether they received active treatment or placebo after every treatment session, not only after the last session. However, Park et al. [62] described potential problems associated with this strategy: “Frequent questioning of the participants may trigger their curiosity about their treatment group. This may affect their whole attitude towards the study, influencing factors such as noncompliance and drop-out that could make the treatment effects observed questionable. This can jeopardize the internal validity of the trial, and its practicality” (p. 121). Park et al. [62] concluded that it is thus “sensible to conduct such surveys at the end of study”, as Folmer et al. [28] did for their clinical trial. However, adequate blinding remains problematic for many rTMS studies.

### 3.4. Characteristics of the Study Population

- Study Population Size

Many review articles and other publications on rTMS for tinnitus end with a similar recommendation: larger, multi-site clinical trials of this treatment method are needed before it can achieve its clinical potential [16,17,18,19,28,29,30,31]. Unfortunately, funding agencies that are capable of supporting such investigations seem reluctant to do so. Perhaps this is due to some negative findings that have been published [32,33]. However, the large number of publications that report positive results for rTMS treatment of tinnitus should be a basis for optimism about the method. These positive reports should encourage funding agencies to support additional investigations involving larger study populations. Only then can some of these issues be addressed and resolved.

- Etiology of Tinnitus

Most cases of subjective tinnitus are associated with hearing loss or damage to the patient’s auditory system [5]. However, subgroups of etiologies exist, such as hearing loss due to aging, genetic factors, loud noise exposure, or disease, as well as auditory damage due to head trauma, infections, whiplash injury, or ototoxic agents. Some prescription or recreational drugs can also contribute to hearing loss and/or tinnitus. Few studies of rTMS for tinnitus have analyzed the etiologies of participants’ tinnitus to discern any patterns associated with treatment outcomes. It is likely that larger patient populations are required for such analyses to yield useful results.

- Duration of Tinnitus

It is not clear how tinnitus duration affects the efficacy of rTMS for the condition. In Landgrebe’s study [33], tinnitus duration averaged 6.2 years for the active rTMS group and 8.1 years for the sham group. In Folmer’s study [28], the mean tinnitus duration was 13 years for the active rTMS group and 18 years for the sham group. Landgrebe et al. [33] postulated that a shorter tinnitus duration would be a positive predictor for treatment outcome. However, Folmer et al. [28] reported that participants in the active rTMS group who had experienced tinnitus for at least 11 years exhibited greater reductions in tinnitus severity compared with participants who had experienced tinnitus for less than10 years.

- Tinnitus Severity

It is likely that baseline (pre-treatment) levels of tinnitus severity reported by rTMS study participants affect the treatment outcomes. For example, participants in the clinical trial by Folmer et al. [28] exhibited these mean baseline values for the tinnitus functional index (TFI) scores:

Responders in the Active rTMS group: 51.4 std dev 18.4 (n = 18 of 32)
Responders in the Sham rTMS group: 55.0 std dev 22.2 (n = 7 of 32)
Non-Responders in the Active rTMS group: 36.3 std dev 17.6 (n = 14 of 32)
Non-Responders in the Sham rTMS group: 36.6 std dev 20.7 (n = 25 of 32)

In this study, the number and percentage of treatment responders in the active stimulation group were greater than the number and percentage of treatment responders in the sham rTMS group. However, it is apparent that study participants in either group with higher baseline TFI scores were more likely to be treatment responders than study participants with lower baseline TFI scores. This result might reflect “regression to the mean” or the fact that participants with lower baseline TFI scores had less room for improvement. In any case, tinnitus severity levels at baseline should be considered when evaluating and analyzing the treatment outcomes.

- Age of Study Participants

**Figure 3:**
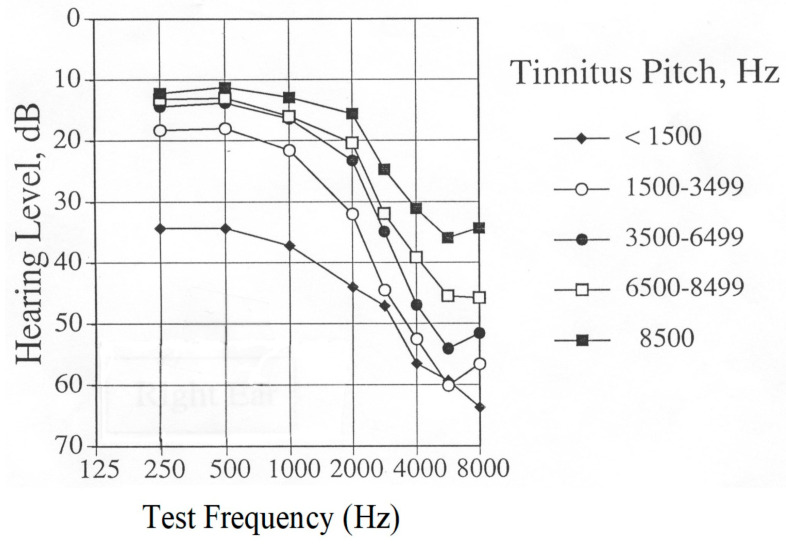
Grand averaged audiograms of tinnitus patients, grouped by pitch of matched tinnitus perception (approximately 200 patients per group).

It is not clear how the age of tinnitus patients affects their response to rTMS treatment. People acquire more hearing loss as their age increases, and the pitch of tinnitus decreases with greater hearing loss (see Figure 3). In addition, the duration of tinnitus increases with the increased age of the study populations. The mean age of participants in Landgrebe’s study [33] was approximately 49 years; the mean age of participants in Folmer’s clinical trial [28] was 60.5 years. However, it is not known if this difference affected study outcomes.

- Co-Occurring Conditions

Research has demonstrated that the severity of tinnitus is positively correlated with the severity of depression [1], insomnia [2], anxiety [3], and obsessive compulsiveness [7] experienced by individuals. Because rTMS is used clinically to treat depression and anxiety disorders, it is likely to help some tinnitus patients who also experience these co-conditions. This might be especially pertinent for dual site rTMS that delivers stimulation to both the frontal and temporal lobes, because frontal lobe stimulation is routinely used for psychiatric applications/treatment. A course of rTMS also improves sleep patterns for some patients, which can contribute to reductions in tinnitus severity [28,63].

- Hearing Sensitivity of Participants

It is not clear how the hearing sensitivity of participants might affect rTMS treatment for tinnitus, because most published studies do not provide this information. Landgrebe et al. [33] recruited subjects with the aim of minimizing hearing loss exhibited by participants. Folmer et al. [28] did not exclude participants based on hearing sensitivity. In fact, Theodoroff and Folmer [64] reported that a deaf individual with tinnitus did not benefit from a course of rTMS treatment. Patients with greater levels of hearing loss tend to perceive lower-pitched tinnitus compared with patients with better hearing sensitivity (see Figure 3 below). However, it is not known how these factors interact with rTMS. However, clinicians or technicians who administer rTMS and patients who receive the treatment should wear hearing protection during rTMS sessions [65]. The study by Folmer et al. [28] demonstrated that the hearing sensitivity of participants—who all wore ear plugs during rTMS sessions—did not change significantly after the course of treatment compared with the pre-treatment levels. 

### 3.5. Outcome Measures and Follow Up

Various outcome measures have been used in rTMS tinnitus treatment studies: Landgrebe et al. [33] used the tinnitus questionnaire (TQ), Noh et al. [42] used the tinnitus handicap inventory (THI) [66], and Folmer et al. [28] used the tinnitus functional index (TFI) [67]. Because the TQ and the THI have only three response options per item (compared to 11 for the TFI), it is likely that TFI has greater sensitivity for treatment-related changes in tinnitus severity compared with those instruments. 

In addition to measures of tinnitus severity, assessments of co-conditions such as depression, anxiety, insomnia, and quality of life should also be used in rTMS studies for tinnitus. Improvements in these conditions are often associated with reductions in tinnitus severity [63] and improved quality of life for patients.

- Follow up

Long-term follow-up (6–12 months or more) of tinnitus patients who undergo rTMS treatment for tinnitus is vital to determine if post-treatment improvements are sustained over time. This can be accomplished by in-person evaluations or by the administration of questionnaires/surveys that patients complete on their own time at home. Folmer et al. [28] reported that improvements in tinnitus severity (as measured by TFI scores) experienced by rTMS treatment responders were sustained during a 26-week follow-up period. 

## 4. Discussion

In their review of rTMS studies for the treatment of tinnitus, Soleimani et al. [16] concluded that evidence exists for the clinical efficacy of rTMS. However, Soleimani et al. [16] cautioned that the high variability in the study design and reported outcomes underscore the need to replicate findings with a large number of patients and long-term follow-up. Liang et al. [17] conducted a systematic review and meta-analysis of rTMS for tinnitus and stated that the method demonstrates effectiveness. However, the authors stated that “more large randomized double-blind multi-centre trials are needed for further verification”. This sentiment has been repeated multiple times over the last 15 years by numerous authors and researchers [18,19,28,29,30,31]. 

Unfortunately, very few large-scale, multi-site clinical trials of rTMS for tinnitus treatment have been conducted. Without such studies, it is impossible to resolve the issues described in this article or to determine which rTMS protocols are most effective for the treatment of tinnitus. Why is there a dearth of large, multi-site clinical trials of rTMS for tinnitus? Based on my personal experience, many funding agencies do not seem interested in investing in this method. This decision might result from a focus on published studies that reported negative findings [32,33], but it is difficult to explain how/why the greater number of studies with positive results continue to be ignored or discounted. Perhaps this has become a vicious cycle: funding agencies are not convinced that rTMS holds promise as a tinnitus treatment because most published studies have involved small sample sizes and their methods/results have not been consistent. So, funding agencies are reluctant to support large clinical trials of rTMS for tinnitus, which would facilitate the refinement and clinical application of this treatment method.

This article is not intended to be a systematic or comprehensive review. Instead, it identifies and discusses issues that need to be addressed in future studies and trials of rTMS for the treatment of tinnitus. However, evidence from numerous published studies indicates that rTMS is an effective treatment for many people who experience chronic and bothersome tinnitus. In some respects, the evidence for the efficacy of rTMS for tinnitus is stronger than the evidence for the efficacy of rTMS treatment for depression [54]. And yet, rTMS is now accepted and used throughout the world as a treatment for major depression, while rTMS for tinnitus remains underfunded, understudied, and underutilized. It is time to rectify this situation for the benefit of patients who suffer from tinnitus and who would benefit from this non-invasive treatment method.

## Figures and Tables

**Table 1:** Compilation of subjects’ guesses (made immediately after their last rTMS session) regarding which treatment they received.

	Was the Guess Correct?	
Subjects’ Guess	YES	NO
They Received Active rTMS	11 subjects	11 subjects
They Received Placebo rTMS	21 subjects	21 subjects

## References

[1] Folmer R.L., Griest S.E., Meikle M.B., Martin W.H. (1999). Tinnitus severity, loudness and depression. Otolaryngol. Head Neck Surg..

[2] Folmer R.L., Griest S.E. (2000). Tinnitus and insomnia. Am. J. Otolaryngol..

[3] Folmer R.L., Griest S.E., Martin W.H. (2001). Chronic tinnitus as phantom auditory pain. Otolaryngol. Head Neck Surg..

[4] Folmer R.L., Griest S.E. (2003). Chronic tinnitus resulting from head or neck injuries. Laryngoscope.

[5] Folmer R.L., Martin W.H., Shi Y. (2004). Tinnitus: Questions to reveal the cause, answers to provide relief. J. Fam. Pract..

[6] Folmer R.L. (2006). Ringing-in-the-Ears: Hope and Help for Tinnitus Sufferers. Hear. Health.

[7] Folmer R.L., Griest S.E., Martin W.H. (2008). Obsessive-Compulsiveness in a Population of Tinnitus Patients. Int. Tinnitus J..

[8] Folmer R.L., McMillan G.P., Austin D.F., Henry J.A. (2011). Audiometric thresholds and prevalence of tinnitus among male Veterans in the United States: Data from the National Health and Nutrition Examination Survey, 1999–2006. J. Rehabil. Res. Dev..

[9] Folmer R.L. (2012). Implants Can Improve Tinnitus, But Most Offer No Quick Fixes. Hear. J..

[10] Folmer R.L., Theodoroff S.M., Martin W.H., Shi Y.B. (2014). Experimental, Controversial and Futuristic Treatments for Chronic Tinnitus. J. Am. Acad. Audiol..

[11] Theodoroff S.M., Lewis M.S., Folmer R.L., Henry J.A., Carlson K.F. (2015). Hearing Impairment and Tinnitus: Prevalence, Risk Factors, and Outcomes in US Service Members and Veterans Deployed to Iraq and Afghanistan Wars. Epidemiol. Rev..

[12] Folmer R.L., Carroll J.R. (2006). Long-term effectiveness of ear-level devices for tinnitus. Otolaryngol. Head Neck Surg..

[13] Eichhammer P., Langguth B., Marienhagen J., Kleinjung T., Hajak G. (2003). Neuronavigated repetitive transcranial magnetic stimulation in patients with tinnitus: A short case series. Biol. Psychiatry.

[14] Langguth B., Eichhammer P., Wiegand R., Marienhegen J., Maenner P., Jacob P., Hajak G. (2003). Neuronavigated rTMS in a patient with chronic tinnitus. Effects of 4 weeks treatment. Neuroreport.

[15] Theodoroff S.M., Folmer R.L. (2013). Repetitive Transcranial Magnetic Stimulation as a Treatment for Chronic Tinnitus: A Critical Review. Otol. Neurotol..

[16] Soleimani R., Jalali M.M., Hasandokht T. (2016). Therapeutic impact of repetitive transcranial magnetic stimulation (rTMS) on tinnitus: A systematic review and meta-analysis. Eur. Arch. Otorhinolaryngol..

[17] Liang Z., Yang H., Cheng G., Huang L., Zhang T., Jia H. (2020). Repetitive transcranial magnetic stimulation on chronic tinnitus: A systematic review and meta-analysis. BMC Psychiatry.

[18] Chen J.-J., Zeng B.-S., Wu C.-N., Stubbs B., Carvalho A.F., Brunoni A.R., Su K.-P., Tu Y.-K., Wu Y.-C., Chen T.-Y. (2020). Association of Central Noninvasive Brain Stimulation Interventions with Efficacy and Safety in Tinnitus Management: A Meta-analysis. JAMA Otolaryngol. Head Neck Surg..

[19] Yin L., Chen X., Lu X., An Y., Zhang T., Yan J. (2021). An updated meta-analysis: Repetitive transcranial magnetic stimulation for treating tinnitus. J. Int. Med. Res..

[20] Arnold W., Bartenstein P., Oestreicher E., Romer W., Schwaiger M. (1996). Focal metabolic activation in the predominant left auditory cortex in patients suffering from tinnitus: A PET study with [^18^F]deoxyglucose. ORL.

[21] Lockwood A.H., Salvi R.J., Coad M.L., Towsley M.L., Wach D.S., Murphy B.W. (1998). The functional neuroanatomy of tinnitus: Evidence for limbic system links and neuroplasticity. Neurology.

[22] Folmer R.L. (2007). Lateralization of Neural Activity Associated with Tinnitus. Neuroradiology.

[23] Maudoux A., Lefebvre P., Cabay J.E., Demertzi A., Vanhaudenhuyse A., Laureys S., Soddu A. (2012). Auditory resting-state network connectivity in tinnitus: A functional MRI study. PLoS ONE.

[24] Schmidt S.A., Akrofi K., Carpenter-Thompson J.R., Husain F.T. (2013). Default mode, dorsal attention and auditory resting state networks exhibit differential functional connectivity in tinnitus and hearing loss. PLoS ONE.

[25] Elgoyhen A.B., Langguth B., De Ridder D., Vanneste S. (2015). Tinnitus: Perspectives from human neuroimaging. Nat. Rev. Neurosci..

[26] Chen Y.C., Xia W., Chen H., Feng Y., Xu J.J., Gu J.P., Salvi R., Yin X. (2017). Tinnitus distress is linked to enhanced resting-state functional connectivity from the limbic system to the auditory cortex. Hum. Brain Mapp..

[27] Husain F.T. (2016). Neural networks of tinnitus in humans: Elucidating severity and habituation. Hear Res..

[28] Folmer R.L., Theodoroff S.M., Casiana L., Shi Y.B., Griest S.E., Vachhani J. (2015). Repetitive Transcranial Magnetic Stimulation Treatment for Chronic Tinnitus: A Randomized Clinical Trial. JAMA Otolaryngol. Head Neck Surg..

[29] Langguth B., de Ridder D., Dornhoffer J.L., Eichhammer P., Folmer R.L., Frank E., Fregni F., Gerloff C., Khedr E., Kleinjung T. (2008). Controversy: Does repetitive transcranial magnetic stimulation/ transcranial direct current stimulation show efficacy in treating tinnitus patients?. Brain Stimul..

[30] Piccirillo J.F. (2016). Transcranial Magnetic Stimulation for Chronic Tinnitus. JAMA.

[31] Mennemeier M., George M. (2017). The Case for a Definitive Multisite, Randomized Clinical Trial of Repetitive Transcranial Magnetic Stimulation for Tinnitus. JAMA Otolaryngol. Head Neck Surg..

[32] Piccirillo J.F., Kallogjeri D., Nicklaus J., Wineland A., Spitznagel E.L., Vlassenko A.G., Benzinger T., Mathews J., Garcia K.S. (2013). Low-Frequency Repetitive Transcranial Magnetic Stimulation to the Temporoparietal Junction for Tinnitus: Four-Week Stimulation Trial. JAMA Otolaryngol. Head Neck Surg..

[33] Landgrebe M., Hajak G., Wolf S., Padberg F., Klupp P., Fallgatter A.J., Polak T., Höppner J., Haker R., Cordes J. (2017). 1-Hz rTMS in the treatment of tinnitus: A sham-controlled, randomized multicenter trial. Brain Stimul..

[34] Schoisswohl S., Langguth B., Weber F.C., Abdelnaim M.A., Hebel T., Mack W., Schecklmann M. (2023). One way or another: Treatment effects of 1 Hz rTMS using different current directions in a small sample of tinnitus patients. Neurosci. Lett..

[35] Pell G.S., Zangen A., Roth Y., Shachar H., Isserles M., Barnea-Ygael N. (2023). Behavioral and Functional Brain Activity Alterations Induced by TMS Coils with Different Spatial Distributions. eNeuro.

[36] Ilmoniemi R.J., Deng Z.-D., Gomez L., Koponen L.M., Nieminen J.O., Peterchev A.V., Epstein C.M., Wassermann E.M., Peterchev A.V., Ziemann U., Lisanby S.H., Siebner H.R., Walsh V. (2021). Transcranial magnetic stimulation coils. The Oxford Handbook of Transcranial Stimulation.

[37] Langguth B., Zowe M., Landgrebe M., Sand P., Kleinjung T., Binder H., Hajak G., Eichhammer P. (2006). Transcranial Magnetic Stimulation for the treatment of tinnitus: A new coil positioning method and first results. Brain Topogr..

[38] Khedr E.M., Rothwell J.C., Ahmed M.A., El-Atar A. (2008). Effect of daily repetitive transcranial magnetic stimulation for treatment of tinnitus: Comparison of different stimulus frequencies. J. Neurol. Neurosurg. Psychiatry.

[39] Noh T.-S., Rah Y.-C., Kyong J.S., Kim J.S., Park M.K., Lee J.H., Oh S.H., Chung C.K., Suh M.-W. (2017). Comparison of treatment outcomes between 10 and 20 EEG electrode location system-guided and neuronavigation-guided repetitive transcranial magnetic stimulation in chronic tinnitus patients and target localization in the Asian brain. Acta Oto-Laryngol..

[40] Langguth B., Landgrebe M., Frank E., Schecklmann M., Sand P.G., Vielsmeier V., Hajak G., Kleinjung T. (2014). Efficacy of different protocols of transcranial magnetic stimulation for the treatment of tinnitus: Pooled analysis of two randomized controlled studies. World J. Biol. Psychiatry.

[41] Formanek M., Migalova P., Krulova P., Bar M., Jancatova D., Zakopcanova-Srovnalova H., Tomášková H., Zeleník K., Komínek P. (2018). Combined transcranial magnetic stimulation in the treatment of chronic tinnitus. Ann. Clin. Transl. Neurol..

[42] Noh T.-S., Kyong J.-S., Park M.K., Lee J.H., Oh S.H., Suh M.-W. (2020). Dual-site rTMS is More Effective than Single-site rTMS in Tinnitus Patients: A Blinded Randomized Controlled Trial. Brain Topogr..

[43] Poeppl T.B., Schecklmann M., Sakreida K., Landgrebe M., Langguth B., Eickhoff S.B. (2021). Prediction of response to repetitive transcranial magnetic stimulation in phantom sounds based on individual brain anatomy. Brain Commun.

[44] Marder K.G., Cho J., Chincanchan R., Wilson A.C., Corlier J., Krantz D.E., Ginder N.D., Lee J.C., Wilke S.A., Tadayonnejad R. (2022). Sequential Prefrontal and Temporoparietal Repetitive Transcranial Magnetic Stimulation (rTMS) for Treatment of Tinnitus with and Without Comorbid Depression: A Case Series and Systematic Review. Front. Neurol..

[45] Frank G., Kleinjung T., Landgrebe M., Vielsmeier V., Steffenhagen C., Burger J., Frank E., Vollberg G., Hajak G., Langguth B. (2010). Left temporal low frequency rTMS for the treatment of tinnitus: Clinical predictors of treatment outcome—A retrospective study. Eur. J. Neurol..

[46] Chen R., Classen J., Gerloff C., Celnik P., Wassermann E.M., Hallett M., Cohen L.G. (1997). Depression of motor cortex excitability by low-frequency transcranial magnetic stimulation. Neurology.

[47] Ring A., Crowder C., Wyer S.L., Phillips B. (2020). A Chart Review to Assess the Response of Veterans Suffering from Tinnitus to Alpha Burst Transcranial Magnetic Stimulation. Int. Tinnitus J..

[48] Hong S.-M., Kim S.-K., Seo M.-Y., Kang S.-Y. (2021). Multiple Daily Rounds of Theta-Burst Stimulation for Tinnitus: Preliminary Results. Medicina.

[49] Godbehere J., Sandhu J., Evans A., Twigg V., Scivill I., Ray J., Barker A. (2019). Treatment of Tinnitus Using Theta Burst Based Repetitive Transcranial Magnetic Stimulation -- A Single Blinded Randomized Control Trial. Otol. Neurotol..

[50] Kleinjung T., Eichhammer P., Langguth B., Jacob P., Marienhagen J., Hajak G., Wolf S.R., Strutz J. (2005). Long-term effects of repetitive transcranial magnetic stimulation (rTMS) in patients with chronic tinnitus. Otolaryngol. Head Neck Surg..

[51] Rossi S., De Capua A., Ulivelli M., Bartalini S., Falzarano V., Filippone G., Passero S. (2007). Effects of repetitive transcranial magnetic stimulation on chronic tinnitus: A randomised, cross over, double blind, placebo-controlled study. J. Neurol. Neurosurg. Psychiatry.

[52] Plewnia C., Bartels M., Gerloff C. (2003). Transient suppression of tinnitus by transcranial magnetic stimulation. Ann Neurol..

[53] Herbsman T., Avery D., Ramsey D., Holtzheimer P., Wadjik C., Hardaway F., Haynor F., George M.S., Nahas Z. (2009). More lateral and anterior prefrontal coil location is associated with better repetitive transcranial magnetic stimulation antidepressant response. Biol. Psychiatry.

[54] Yesavage J.A., Fairchild J.K., Mi Z., Biswas K., Davis-Karim A., Phibbs C.S., Forman S.D., Thase M., Williams L.M., Etkin A. (2018). Effect of repetitive transcranial magnetic stimulation on treatment-resistant major depression in US Veterans: A randomized clinical trial. JAMA Psychiatry.

[55] Cole E.J., Phillips A.L., Bentzley B.S., Stimpson K.H., Nejad R., Barmak F., Veerapal C., Khan N., Cherian K., Felber E. (2022). Stanford Neuromodulation Therapy (SNT): A Double-Blind Randomized Controlled Trial. Am. J. Psychiatry.

[56] Dobie R.A. (1999). A review of randomized clinical trials in tinnitus. Laryngoscope.

[57] Folmer R.L., Carroll J.R., Rahim A., Shi Y., Martin W.H. (2006). Effects of Repetitive Transcranial Magnetic Stimulation (rTMS) on Chronic Tinnitus. Acta Otolaryngol..

[58] Anders M., Dvorakova J., Rathova L., Havrankova P., Pelcova P., Vaneckova M., Jech R., Holcat M., Seidl Z., Raboch J. (2010). Efficacy of repetitive transcranial magnetic stimulation for the treatment of refractory chronic tinnitus: A randomized, placebo controlled study. Neuro Endocrinol. Lett..

[59] Khedr E.M., Rothwell J.C., El-Atar A. (2009). One-year follow up of patients with chronic tinnitus treated with left temporoparietal rTMS. Eur. J. Neurol..

[60] Vanneste S., Plazier M., van der Loo E., Ost J., Van de Heyning P., De Ridder D. (2010). Burst transcranial magnetic stimulation: Which tinnitus characteristics influence the amount of transient tinnitus suppression?. Eur. J. Neurol..

[61] Keller D.L. (2015). Assessment of Blinding in a Tinnitus Treatment Trial. JAMA Otolaryngol. Head Neck Surg..

[62] Park J., Bang H., Cañette I. (2008). Blinding in clinical trials, time to do it better. Complement. Ther. Med..

[63] Folmer R.L. (2002). Long-term reductions in tinnitus severity. BMC Ear Nose Throat Disord..

[64] Theodoroff S.M., Folmer R.L. (2015). Experimental Use of Transcranial Magnetic Stimulation (TMS) to Treat Tinnitus in a Deaf Patient. Clin. Med. Rev. Case Rep..

[65] Folmer R.L., Theodoroff S.M. (2017). Hearing Protective Devices Should Be Used by Recipients of Repetitive Transcranial Magnetic Stimulation. J. Clin. Neurophysiol..

[66] Newman C.W., Jacobson G.P., Spitzer J.B. (1996). Development of the Tinnitus Handicap Inventory. Arch Otolaryngol. Head Neck Surg..

[67] Meikle M.B., Henry J.A., Griest S.E., Stewart B.J., Abrams H.B., McArdle R., Myers P.J., Newman C.W., Sandridge S., Turk S.C. (2012). The Tinnitus Functional Index: A new clinical measure for chronic, intrusive tinnitus. Ear Hear..

